# Safety and long-term efficacy of autologous hematopoietic cell transplantation for patients with systemic sclerosis

**DOI:** 10.3389/fmed.2025.1527779

**Published:** 2025-07-14

**Authors:** Eleni Gavriilaki, Despina Mallouri, Ioannis Batsis, Zoi Bousiou, Anna Vardi, Nikolaos Spyridis, Georgios Karavalakis, Alkistis kyra Panteliadou, Panagiotis Dolgyras, Christos Varelas, Vlasios I. Alevizopoulos, Panagiotis G. Asteris, Panayiotis Vlachoyiannopoulos, Damianos Sotiropoulos, Petros P. Sfikakis, Ioanna Sakellari

**Affiliations:** ^1^Hematology Department and Bone Marrow Transplant (BMT) Unit, G. Papanicolaou Hospital, Thessaloniki, Greece; ^2^2nd Propedeutic Department of Internal Medicine, Hippocration Hospital, Aristotle University of Thessaloniki, Thessaloniki, Greece; ^3^German Oncology Center, Limassol, Cyprus; ^4^Computational Mechanics Laboratory, School of Pedagogical and Technological Education, Athens, Greece; ^5^Department of Pathophysiology, School of Medicine, National and Kapodistrian University of Athens, Greece; Institute for Autoimmune Systemic and Neurologic Diseases, Athens, Greece; ^6^1st Department of Propaedeutic and Internal Medicine, Medical School, Joint Academic Rheumatology Program, National and Kapodistrian University of Athens, Athens, Greece

**Keywords:** cell therapy, hematopoietic cell transplantation, systemic sclerosis, scleroderma, treatment-related mortality

## Abstract

Autologous hematopoietic cell transplantation (HCT) has been introduced for patients with severe systemic sclerosis (SSc). We aimed to assess the safety and long-term efficacy of HCT modality for severe SSc, refractory to conventional therapy, in 17 patients who were referred to our – The Joint Accreditation Committee of the International Society for Cellular Therapy (ISCT) and the European Group for Blood and Marrow Transplantation (EBMT)-accredited Unit from 2005 to 2024. Peripheral blood stem cells were collected using cyclophosphamide and GCSF. An immunoablative conditioning regimen of cyclophosphamide and anti-thymocyte globulin was administered. Disease assessments were done before and after mobilization treatment and post-transplant, focusing on skin sclerosis, pulmonary function, cardiac involvement, gastrointestinal manifestations, the necessity for additional immunosuppressive therapy, and overall patient well-being. Before transplantation, 13/17 (76%) of the patients had diffuse skin involvement with a mean mRSS of 31 (2–49), 2/17 (12%) had pulmonary hypertension, and 14/17 (82%) had gastrointestinal manifestations. The median follow-up period was 9.1 (0. 5–14. 3) years. Improvement of skin sclerosis was observed, with a decrease in mRSS before transplantation from 31 (2–49) to 7 (2–22) post-HCT. Lung function remained stable in 8/15 (53%) patients, improved in 5/15 (33%), and deteriorated in 2/15 (13%). Gastrointestinal manifestations were improved in 12/14 (86%) patients, while all patients (16/16, 100%) reported a great impact on their quality of life. Ten out of the 16 (63%) patients were free of immunosuppressive drugs after the HCT. Overall survival was 16/17 (94.2%). Concerning TRM, there was one (1/17, 5.8%) death early post-transplant. In this specific cohort of selected patients with severe SSc refractory to immunosuppressive medications, autologous HCT led to improvements in the outcomes assessed.

## 1 Introduction

Systemic sclerosis (SSc) is an immune-mediated rheumatic disease characterized by three main dysfunctions, namely autoimmune responses, vascular damage, and fibrosis of the skin and internal organs, including the lungs, heart, kidney, and gastrointestinal tract (1), with a heterogeneous clinical appearance. Pre-scleroderma, sine scleroderma, limited, and diffuse types are distinct forms of the disease. Patients with ‘pre-scleroderma’ have some characteristics of systemic sclerosis (isolated Raynaud’s phenomenon, puffy fingers, specific autoantibodies or SSc-associated changes to the capillaries) and their presence predetermines to some extent the progression to systemic sclerosis (2). There is also another subcategory of patients lacking definite skin involvement but with major internal organ-based manifestations. The evolution of this type, which is called “sine scleroderma,” is similar to those with limited cutaneous systemic sclerosis (lcSSc) (3). The two main subtypes are the limited and diffuse cutaneous systemic sclerosis (dcSSc), depending on the extent of skin involvement. The first is characterized by fibrosis of skin distal to the elbows and/or knees and possible skin thickening on the face and the neck without truncal involvement. The latter affects the skin both distal and proximal to the knees and/or elbows with probable truncal involvement. Patients with dcSSc present more frequently with tendon friction rubs, interstitial lung disease, heart or gastrointestinal disease (both of the esophagus and the more distal digestive tract), and Scl70 autoantibodies, while patients with lcSSc more frequently present with severe Raynaud’s phenomenon, pulmonary hypertension, esophageal disease without distal digestive tract involvement, and anticentromere antibodies (4).

Data show that mortality rates of SSc have steadily declined in the past two decades, although systemic sclerosis patients still experience a significant burden of disease (5, 6). The overall prognosis of the two main types of systemic sclerosis (diffuse and limited) is different (7). A recent meta-analysis that does not include recipients of hematopoietic cell transplantation reports high standardized mortality ratio (SMR) for patients with the diffuse type (3.7–6.1) and a mean standardized mortality ratio of 4.7, while SMR for the limited type is estimated at 2.04 and the overall SMR for systemic sclerosis at 2.72 (8). Ten-year survival rates from the time a patient is diagnosed with systemic sclerosis has been estimated to be between 55 and 73% (9). The leading causes of SSc mortality are considered to be complications related to interstitial lung disease (ILD) and cardiac involvement, including pulmonary arterial hypertension (10). High mortality rates underscore the need for novel, more effective therapeutic modalities, while the importance to diagnose the presence of systemic sclerosis early, for a favorable response to targeted therapies and the prevention of irreversible organ damage, is paramount (11, 12).

On top of non-HCT therapies, autologous hematopoietic stem cell transplantation is a promising therapeutic approach (13). HCT eradicates the autoimmune system, replacing it with a new immune repertoire with tolerance to autoantigens and long-lasting regulation. Regarding autologous HCT for autoimmune disorders, in 2023, 51 autologous HCT procedures were performed for the treatment of systemic sclerosis, which is second in prominence only to multiple sclerosis (*n* = 419), for which, among other autoimmune diseases, autologous HCT is mainly indicated (14).

The development of autologous HCT as a therapeutic modality for systemic sclerosis has overcome major barriers in the last 25 years, such as morbidity and treatment-related mortality rates that were too high for an autoimmune disease. Best clinical practice with a very strict and careful patient selection, as well as optimal early timing of the transplant procedure, emerged as a critical value. Many organizations have recommended autologous HCT for early severe diffuse cutaneous SSc patients. The European Group for Blood and Marrow Transplantation in 2012 (15), with a grade II strength recommendation, the American Society for Transplantation and Cellular Therapy with developmental indication (D) in 2015 (16), the European Society for Blood and Marrow Transplantation (EBMT) with a grade I level of evidence in 2022 (17), and the European League Against Rheumatism in 2023 have all recommended autologous HCT for selected patients with early severe dcSSc and poor prognosis in the absence of advanced cardiorespiratory involvement (18). The Brazilian Society of Rheumatology has also recommended it for the treatment of refractory cases of SSc (19), as has the British Society for Rheumatology, who recommend auto-HCT in selected dcSSc patients where benefit is likely to be greater than treatment-related risk (level of evidence 1B) (20). While HCT is not a therapeutic option for some high-risk patients with poor cardiopulmonary status, recently discovered medications seem to be less cardiotoxic (21). New approaches implement smaller neutropenic intervals (22) and patients who do not meet the current criteria seem to response well to HCT (23). In other words, HCT seems to be an effective approach to an otherwise lethal disease (24).

The present study aims to evaluate the safety and long-term efficacy of HCT modality for severe SSc patients at a single expert center, highlighting the strengths and limitations of this approach.

## 2 Materials and methods

### 2.1 Study design

This study is a retrospective, single-center study. The aim of the study was to assess the safety and long-term efficacy of autologous HCT in patients with severe systemic sclerosis. All HCT procedures took place in the JACIE-accredited Hematopoietic Cell Transplantation Unit, in the G. Papanicolaou Hospital, Thessaloniki, Greece, between 2005 and 2024. Patients were eligible for the HCT procedure if they fulfilled the 1980 classification criteria of the American College of Rheumatology for SSc (25), had skin sclerosis characterized by modified Rodnan skin score (mRSS) ≥ 15 in parallel with cardiac, renal, or pulmonary involvement, or if the disease course was refractory to any conventional therapy. Patients with severe heart failure (left ventricular ejection fraction below 50%), pulmonary hypertension (systolic pulmonary artery pressure > 50 mmHg), severe respiratory failure (DLCO < 40%), renal failure (CrCl < 40 mL/min/m^2^), or an active ongoing infection were considered not eligible for HCT. In total, 21 patients with severe systemic sclerosis were referred to our center for HCT, however, only 17 were eligible for the procedure. All eligible patients underwent autologous HCT.

The local ethics committee of G. Papanicolaou Hospital approved this study (No. 187/2016), which was conducted according to the Declaration of Helsinki. All patients gave their written informed consent to participate in the study.

### 2.2 Patients’ evaluation

Each patient was thoroughly evaluated prior to transplantation (day 0 was defined as the time of infusion of stem cells) to determine baseline status and identify possible exclusionary conditions. Laboratorial assessment included blood and urine samples for complete blood counts, biochemical profile, urine analysis, creatinine clearance, proteinuria, pregnancy tests, infectious serologies, and the presence or absence of autoantibodies. Skin sclerosis (mRSS) evaluation was based on clinical examination, cardiac functionality was assessed by cardiac magnetic resonance imaging (MRI) and echocardiography, and pulmonary arterial hypertension was diagnosed based on the findings of HRCT. Moreover, pulmonary status was evaluated with the use of HRCT and the assessment of FEV1 and DLCO. Lastly, patient-reported outcomes regarding functional ability and quality of life were collected.

### 2.3 Autologous hematopoietic stem cell transplantation

The first phase of autologous HCT consists of the mobilization of hematopoietic progenitor cells from the bone marrow into the peripheral blood through the infusion of cyclophosphamide at a dose of 4 gr/m^2^ and G-CSF. The next step is the apheresis and cryopreservation of the hematopoietic progenitor cells. The second phase of autologous HCT is characterized by intense immunoablation with an immunoablative regimen that consists of cyclophosphamide (50 mg/kg/d for 4 days) and anti-thymocyte-globulin (Rabbit ATG; 2.5 mg/kg/d for 3 days). Lastly, the previously collected cells are infused. The target CD34 + number was 4 × 10^6^, the standard target according to JACIE accreditation.

### 2.4 Endpoints

The main goal of this study was to examine the safety of this procedure. Early treatment-related mortality was defined as any death during the 100 days following transplant for which HCT was the major causal influencing factor.

Secondary endpoints were overall survival, the need for additional immunosuppressive drugs post-transplant, and the response to treatment. The response to treatment was defined as a > 25% improvement in mRSS, a subjective report of improvement in gastrointestinal symptoms, and an improvement in lung function, which was categorized as follows: “no change” for 0–9% change of FEV1 or DLCO, “improvement” for increases of ≥ 10%, or “deterioration” for decreases of ≥ 10%. Descriptive statistical analysis was performed by SPSS version 22. The limited patient population did not allow for further statistical comparison.

## 3 Results

A total of 17 patients underwent auto-HCT, 14 women (82%) and 3 men (18%). The median age was 34 (range 21–50) years with median disease onset 3 (2–13) years prior to the transplantation. Before HCT, skin involvement was detectable in 13 (13/17, 76%) patients with a median mRSS of 31 (range 2–49). Two patients (2/17, 12%) were also diagnosed with pulmonary arterial hypertension. Median DLCO was 61.0% (range 42–95) and median FEV1 was 78.0% (range 56–98) of the respective target values. Gastrointestinal manifestations were found in 14 of 17 patients (82%). In Table 1, the baseline (pre-transplant) characteristics of all adult transplanted patients are presented. The median follow-up period after HCT was 9.1 (0. 5–14. 3) years.

**Table 1:** Baseline patient characteristics.

Baseline patient characteristics	*N* = 17
Gender, *n* (%)	
Female	14 (82)
Male	3 (18)
Median age years (range)	34 (21–50)
Median disease duration years (range)	3 (2–13)
Mean number of prior administrations of immunosuppressive therapy in each patient (95% CI)	4 (2–7)
Number of patients who received cyclophosphamide infusions, *n* (%)	13 (76)
Mean overall cyclophosphamide dose for each patient, gr (95% CI)	6 (2.5–12)
Mean modified Rodnan skin score (before transplant) (95% CI)	31 (2–49)
Patients with mRSS < 15 and severe lung disease, *n* (%)	4 (23)
Patients with gastrointestinal involvement, *n* (%)	14 (82)
Lung involvement,	
Mean FEV1%	78 (56–98)
Mean DLCO%	61 (42–95)
Patients with pulmonary arterial hypertension, *n* (%)	2 (12)
Mean ejection fraction%,	70 (60–75)
Number of patients with ECOG < 3, *n* (%)	17 (100)

DLCO, diffusing capacity of the lungs for carbon monoxide; ECOG, Eastern Cooperative Oncology Group; FEV1, forced expiratory volume in 1 s; mRSS: Mean Modified Skin Score.

After auto-HCT, the mean period for the neutrophil engraftment was 9 (7–14) days, for the platelet engraftment was 9 (5–13) days, and the mean time for discharge from the unit was 14 (13–38) days.

### 3.1 Immediate transplant outcomes

Two (2/17, 12%) patients presented pneumothorax as a complication of central venous catheter insertion. Fever during the neutropenic phase of auto-HCT was noted in 10 (10/17, 58%) patients. Regarding infections, 3 (3/17, 17%) patients presented lower respiratory tract infections and another (1/17, 5.8%) patient exhibited BK polyomavirus–associated hemorrhagic cystitis. After engraftment, cytomegalovirus (CMV) reactivation was detected in two (2/17, 12%) patients, Epstein Barr reactivation in 4 (4/17, 23%) patients, and Herpes Zoster reactivation in one (1/17, 5.8%) patient. Table 2 presents the complications that HCT recipients experienced during follow-up.

**Table 2:** Complications and transplant-related events after transplantation.

Complications and transplant-related events after transplantation	*n* (%)
Pneumothorax during central venous catheter placement procedure	2 (12)
Neutropenic fever	10 (63)
Lower respiratory tract infection	3 (17)
Polyomavirus-induced hemorrhagic cystitis	1 (5.8)
CMV reactivation	2 (12)
EBV reactivation	4 (23)
Herpes Zoster	1 (5.8)
Transplant-related death (total) Sepsis, Multiple organ dysfunction syndrome	1 (5.8)

CMV, Cytomegalovirus; EBV, Epstein Barr Virus.

### 3.2 Survival

There was one (1/17, 5.8%) death that was considered transplant-related at 87 days after the procedure. This patient was heavily pre-treated with severe pulmonary disease (DLCO 49%). The patient died due to infection, sepsis, and multiorgan failure in the intensive care unit (ICU).

### 3.3 Disease outcomes

The median mRSS was significantly improved after the HCT, with the median mRSS being 7 at the last follow-up (2–22). Regarding lung function, DLCO and FEV1 percentages stabilized after HCT in 8 (8/15, 53%) SSc patients, two (2/15, 13%) patients presented more than a 10% decline in FEV1 or DLCO, and 5 (5/15, 33%) patients presented improvements in both FEV1 and DLCO. Gastrointestinal manifestations were improved in 12 (12/14, 86%) patients. All patients (16/16, 100%) reported a great impact on their quality of life. Further immunosuppressive treatment was needed in 6 (6/16, 37%) patients who had disease reactivation after HCT.

## 4 Discussion

In the past 30 years, hematopoietic cell transplantation has been investigated as a treatment for many autoimmune diseases, including systemic sclerosis. Despite the high risk of transplant-related mortality and other complications, HCT is supposed to be the only treatment able to induce long-term, symptom-free remission without the necessity for additional pharmacological management in many refractory autoimmune rheumatic diseases (13).

Interestingly, the first patient reported to be treated with an HCT for an autoimmune disease had systemic sclerosis complicated with pulmonary artery hypertension, who denied lung transplant (26) and remained stable for 20 years after her treatment (27).

Currently, HCT is geared toward patients with severe SSc, but the selection criteria for these patients can go beyond that (4). The efficacy of HCT compared to conventional treatment with immunosuppressive drugs has been highlighted by many trials. Burt et al. via ASSIST (28), van Laar et al. via ASTIS (29), and Sullivan et al. via SCOT (30) demonstrated the superiority of auto-HCT to conventional therapy with monthly cyclophosphamide infusions for early severe systemic sclerosis as far as long-term survival and improvement of lung function and skin fibrosis are concerned. Since then, numerous trials have exhibited the superior efficacy of HCT to cyclophosphamide (24, 31). A small Japanese trial implied the superiority of selective HCT (in which CD34 + cells are chosen for infusion) to the non-selective process (32), van Bijnen et al. reported similar positive results to ASTIS (1), and Henrique-Neto et al. (33) were also in line with these findings supporting HCT as a viable therapeutic choice for severe systemic sclerosis. In accordance with the results of these three prospective randomized trials, Henes et al. (34) confirmed the efficacy of autologous stem cell transplantation for severe systemic sclerosis in a prospective non-interventional study using the ASTIS-regimen, while a recent study assessing long-term outcomes of autologous HCT in comparison to rituximab and traditional immunosuppressive drugs established that auto-HCT is more effective than both in prolonging survival and inducing prolonged remission in patients with severe SSc (31).

Recent data suggest that mortality, quality of life, and skin tightness are among the clinical manifestations that are definitely improved, with high levels of evidence, after the HCT procedure. Lung function (Forced vital capacity, FVC) appears to be mildly improved, while the improvement of pain, interstitial lung disease (as far as the inflammation, not the fibrosis), gastrointestinal symptoms, range of motion of joints, hand grip strength, and exercise capacity is supported by a medium level of evidence. HCT also seems to prevent the development of pulmonary arterial hypertension (PAH) and heart failure (HF), but its effect on established PAH and HF remains unclear. Esophageal motility, myositis, and peripheral neuropathy possibly present an improvement but without a strong level of evidence yet. Conversely, renal function and esophageal dilation appear to be negatively affected (4).

Here, we describe the clinical outcomes of 17 adult SSc patients with a severe form of the disease, refractory to conventional therapy, treated at a single center from 2005 to 2024 under similar transplant protocols. However, our experience in autologous transplantation goes back to 1997 with our pioneering work on multiple sclerosis (35), with our proposed conditioning regimen (BEAM) becoming the main clinical practice.

It seems that the positive outcome of the HCT procedure is correlated with a shorter interval from the diagnosis time to the HCT, as less tissue damage and internal organ impairment has been accumulated over time, so these lesions may still be reversible. Thus, many studies have included patients with disease duration of up to 4 years (28, 29, 36). On the contrary, recent studies that are not in consonance with this limit have reported great results in patients with disease durations of 17 and 15 years, respectively (22, 23). The mean time from diagnosis to HCT for our patients was 3 (2–13) years. As regards the pulmonary function, evaluated by FEV1 and DLCO, 5 patients presented improvement, 8 patients remained stable, and two patients deteriorated, supporting the notion that HCT leads to lung disease stabilization and possibly improvement of lung function. The efficacy of HCT on pulmonary function seems to be directly affected by the baseline cardiac status. In particular, DLCO does improve solely in patients with normal cardiac tests at baseline, while cardiac involvement before transplant predetermines no improvement (37). According to Eyraud et al. (38), who compiled data from randomized studies and four cohort studies, improvement was shown in FVC at 1 or 2 years after HCT. However, it should be stressed that any invasive pretransplant procedure in a critically ill patient to further assess the cardiopulmonary status can significantly aggravate the condition and should be avoided, as we commented in a previous study (39). As far as skin thickness is concerned, its clinical improvement is easily assessed by the modified Rodnan skin score. The mRSS in our study, before transplant, was 31 (2–49), decreasing steadily to a mean score of 7 (2–22) at the last follow-up after transplantation. Numerous studies support this notable decrease of skin thickness for up to 8 years after the procedure (38) and its improvement is considered mRSS reduction of more than 25% (40). Interestingly, mRSS > 24 at baseline appears to be significantly associated with lower progression-free survival (34). Skin involvement in systemic sclerosis has reported to affect both survival and quality of life (41). As regards the improvement of gastrointestinal manifestations, our study demonstrated an obvious benefit (12/14, 86%), which is not strongly noted by other trials or reviews (42). Deterioration of the quality of life is a major aspect of the disease, and every patient is eager for its restoration. The “state of being” of the patients, post the procedure, can be a useful indicator of the transplant outcomes and their perspective in general. In our study, in accordance with the evidence of a recent systematic review (43), all of our recipients, except one who did not survive (16/16, 100%), noticed an overall improvement to their lives.

HCT is not a “panacea” for patients with severe SSc. The likelihood of nonresponse or relapsing (whether manageable or not) and the lack of justified prognostic factors for the 4–17% of patients who experience transplant-related mortality (9), as well as the moderate effects on lung function improvement (44) and the relatively high number of transplant-related complications (45), hamper the curative effect of HCT. Interestingly, most patients who experienced relapse, while unresponsive pre-HCT, successfully resumed immunosuppressive medication, supporting further the notion that HCT leads to a “reset” of the immune system (27). For patients with systemic sclerosis treated with HCT, TRM is considered to be related with the transplant regimen and, thus, cardiotoxicity (46), the optimal regimen for mobilization and conditioning (13), the selection or not of CD34 + graft (34), and the lack of unanimous HCT eligibility criteria. Infections play a substantial role in the success of the transplant in patients with autoimmune diseases, as the immune system of these patients is often substantially weakened in the wake of the chronic use of immunosuppressants and the disease itself. Both the mobilization of stem cells and the conditioning regimen are all associated with an increased risk of infections (15).

Our study reported one transplant-related death (1/17, 5.8%) of a patient who was refractory to immunosuppressives up to 7 times before the HCT and died of infection, sepsis, and multiple organ dysfunction 87 days after the procedure. HCT-related mortality in our series is remarkably low (5.8%) and we have to consider that the HCT procedure was the only treatment choice left in this patient’s therapeutic options. Interestingly, 6 of our patients (6/16, 37%) initiated immunosuppressive therapy after the HCT procedure, at a median time of 2.1 years (0.6–3.2). It is important to stress that the necessity for immunosuppressive drugs after HCT is not uncommon (47), revealing that not all patients achieve a treatment-free remission.

Our study has some methodological limitations. Firstly, this is a single-center open study, so the results may not have the same scientific power as those derived from a multicenter controlled trial. Secondly, there was not a control group treated with conventional immunosuppressive drugs to make the comparison between them or among other studies. Thirdly, our study is retrospective with a small sample size. Lastly, data regarding FVC measurements as well as the autoantibody status, while assessed prior to the auto-HCT, are not available.

High treatment-related mortality, although significantly reduced, remains the Achilles heel of HCT, requiring an assessment of risk versus benefit at an individual level to make an informed decision after appropriate evaluation, investigation, and discussion with each patient. Looking for alternative treatment options with comparable efficacy to HCT, chimeric antigen receptor (CAR) T-cell therapy, as well as new combinations of immunomodulatory drugs (48), have shown promising results. More specifically, the role of CD19-CAR T-cell therapy has been examined (49), with initial evidence showing that this treatment might be effective in severe SSc (50). Finally, an ongoing trial is examining the optimal timing for auto-HCT performance (51). Overall, patient selection criteria is key to successful treatment: patients should have a disease severe enough to proceed to HCT but not too advanced so that they can endure transplant-related toxicity.

## 5 Conclusion

A cure for the severe form of systemic sclerosis has been desired by physicians for many years. Based on our data, autologous HCT is a safe and effective therapy for SSc patients, with durable long-term outcomes. Although many details are yet to be clarified regarding the timing, patients’ eligibility criteria, prognostic factors for the TRM, and a strict framework of guidelines, it seems that a unique opportunity has emerged for people with severe systemic sclerosis and for autoimmune diseases in general.

## Data Availability

The original contributions presented in the study are included in the article/supplementary material, further inquiries can be directed to the corresponding authors.
